# 

**Published:** 1895-03-02

**Authors:** 


					PRICE TWOPENCE. TEJ? IBNll Wl? EXTRA nursing supplement.
No. 440, Vol. XVII,?March 2nd, 1895. Mm. Jlnll JP f\ per Annum?10s- ed-
at the General Port Offloe a. a Newspaper. & U "?ff ^ *?**' P?? 8d?
^ Ditto per Annum, 8s. 8d.,post free.
inWICH HOSPITAL
No. 440. Vol. XVII. WITH EXTRA NURSIN6 8UPPLENIENT. Sattoday, March 2nd, 1895.

				

